# Silicon Differently Affects Apoplastic Binding of Excess Boron in Wheat and Sunflower Leaves

**DOI:** 10.3390/plants12081660

**Published:** 2023-04-15

**Authors:** Jasna Savic, Jelena Pavlovic, Milos Stanojevic, Predrag Bosnic, Ljiljana Kostic Kravljanac, Nina Nikolic, Miroslav Nikolic

**Affiliations:** 1 Faculty of Agriculture, University of Belgrade, Nemanjina 6, 11080 Belgrade, Serbia; 2 Institute for Multidisciplinary Research, University of Belgrade, Kneza Viseslava 1, 11030 Belgrade, Serbia.; 3 Faculty of Sciences and Mathematics, University of Pristina in Kosovska Mitrovica, Lole Ribara 29, 38220 Kosovska Mitrovica, Serbia

**Keywords:** apoplast, boron toxicity, cell wall, leaves, silicon, symplast, sunflower, wheat

## Abstract

Monocots and dicots differ in their boron (B) requirement, but also in their capacity to accumulate silicon (Si). Although an ameliorative effect of Si on B toxicity has been reported in various crops, differences among monocots and dicots are not clear, in particular in light of their ability to retain B in the leaf apoplast. In hydroponic experiments under controlled conditions, we studied the role of Si in the compartmentation of B within the leaves of wheat (*Triticum vulgare* L.) as a model of a high-Si monocot and sunflower (*Helianthus annuus* L.) as a model of a low-Si dicot, with the focus on the leaf apoplast. The stable isotopes ^10^B and ^11^B were used to investigate the dynamics of cell wall B binding capacity. In both crops, the application of Si did not affect B concentration in the root, but significantly decreased the B concentration in the leaves. However, the application of Si differently influenced the binding capacity of the leaf apoplast for excess B in wheat and sunflower. In wheat, whose capacity to retain B in the leaf cell walls is lower than in sunflower, the continuous supply of Si is crucial for an enhancement of high B tolerance in the shoot. On the other hand, the supply of Si did not contribute significantly in the extension of the B binding sites in sunflower leaves.

## 1. Introduction

Boron (B) is a micronutrient for vascular plants with many proposed roles such as sugar transport, the structure of cell walls and membranes, the metabolism of carbohydrates, phenol and RNA, germination, etc., but only its function in cell walls has been fully accepted [1]. Both the deficiency and toxicity of B can decrease crop production, though low-B soils conductive to B deficiency in crops are considerably more widespread than high-B soils [2,3]. Naturally high-B soils (e.g., soils formed from the parent material of marine origin) are most common in arid and semi-arid regions, and are often associated with an excess of Na^+^ and Cl^−^ ions [1,4,5,6]. However, irrigation water high in B, the application of large amounts of municipal compost, B mining and processing, and geothermal activity can be sources of soil contamination with B [1,7]. Boron toxicity in plants is manifested by leaf chlorosis, beginning at the older leaf tips and margins, followed by necrosis [1].

Boron and silicon (Si is a beneficial element for most vascular plants) show considerable chemical similarity (e.g., week Lewis acids and affinity for binding to polyols) and both elements are taken up as uncharged acids (H_3_BO_3_^0^ and H_4_SiO_4_^0^) via nodulin-26-like intrinsic protein-types of aquaporins [8]. Besides the proposed formation of complexes between Si and B in the soil solution [9], their interactions are also possible in the cell walls, because both elements are involved in the cross-linkage of the cell wall structures [10,11]. An ameliorative effect of Si on B toxicity has been reported in various crops such as wheat [12], rice [13], barley [14], pepper [15], cucumber [3], tomato [16], spinach [17], oilseed rape [18], and cotton [19]. The main proposed mechanisms of Si-mediated alleviation of B toxicity are as follows: (a) restricted uptake and/or root-to-shoot transport of B [12,18], (b) redistribution of B between symplastic and apoplastic compartments in leaves [20,21], and (c) enhanced tissue antioxidative capacity [14,22,23].

In principle, monocots, and especially graminaceous species, have a higher capacity for Si shoot accumulation but lower B demand compared to dicots [24]. Celikkol Akcay and Erkan [21] reported the existence of a certain degree of competition within the B transport system favoring Si uptake, which was also the mechanism proposed for oilseed rape grown under excessive B [18]. However, in barley genotypes differing in susceptibility to B toxicity, no competitive interaction was found in the uptake of Si and B [25]. The supply of Si to cucumber exposed to excess B had no effect on total B concentration in leaves, but strongly affected the redistribution of B between the symplast and apoplast [20]. In barley, the short-term simultaneous application of Si and high B upregulated the efflux borate anion transporter genes of the *HvBOR* family [21], which is responsible for the enhancement of B tolerance in the shoot by exporting B from the symplast to the apoplast [26].

The aim of this study was to investigate how Si affects the compartmentation of B within the leaves of wheat and sunflower which differ in their capacity to accumulate Si and B in the shoot. Wheat is a high-Si grass with low shoot B accumulation capacity, while sunflower is a typical low-Si dicot with a five-times-higher shoot concentration of B compared to wheat [24]. Our study tests the hypothesis that the application of Si differently influences the binding capacity of the leaf cell wall for B in these two contrasting species subjected to excess B. 

## 2. Results

### 2.1. Plant Growth and Accumulation of B 

**Figure 1:**
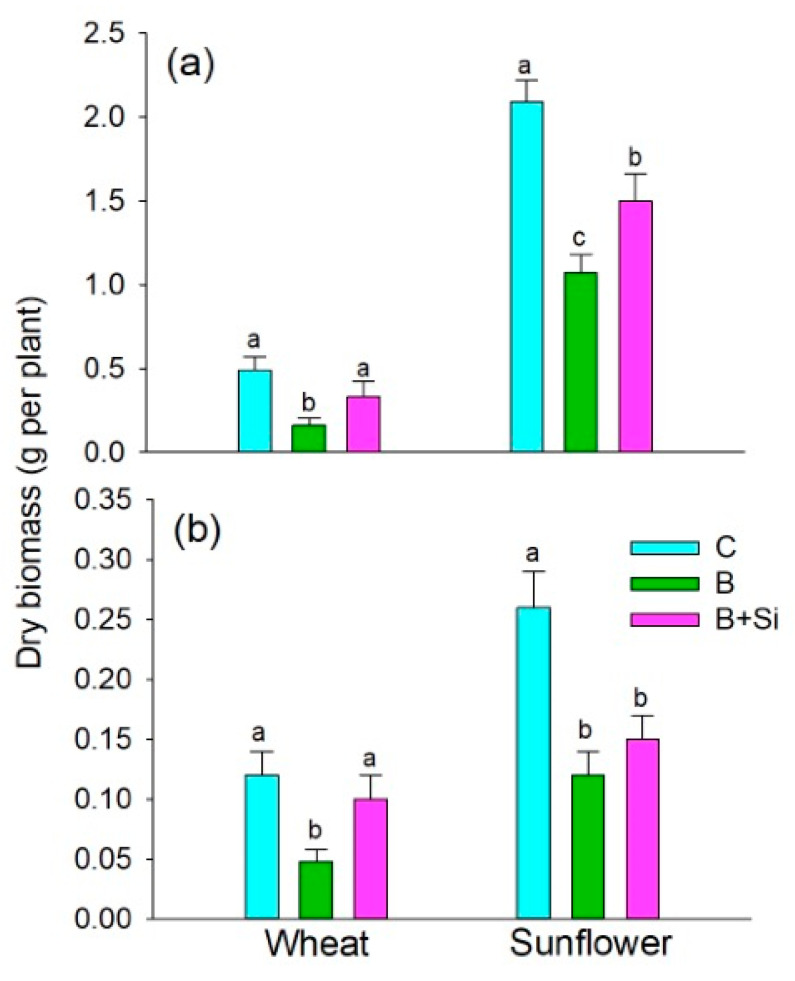
Dry shoot (**a**) and root (**b**) biomass of sunflower and wheat plants. Plants were grown for 14 d in the following treatments: C (control; 1 µM B for wheat and 10 µM B for sunflower), B (500 µM B), and B + Si (500 µM B + 1.5 mM Si). Data are means ± SD (n = 4). Statistically significant differences between treatments for each crop (*p* < 0.05, Tukey’s test) are indicated by different letters.

**Figure 2:**
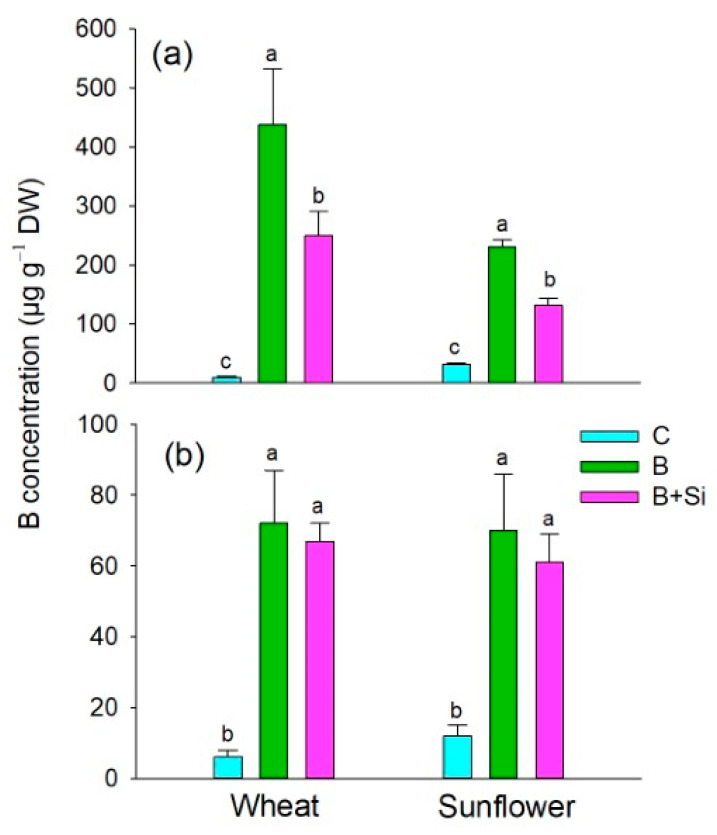
Concentrations of total B in shoot (**a**) and roots (**b**) of sunflower and wheat plants. Plants were grown for 14 d in the following treatments: C (control; 1 µM B for wheat and 10 µM B for sunflower), B (500 µM B), and B + Si (500 µM B + 1.5 mM Si). Data are means ± SD (n = 4). Statistically significant differences between treatments for each crop (*p* < 0.05, Tukey’s test) are indicated by different letters.

The excessive supply of B decreased both the root and shoot growth of wheat and sunflower during two weeks of exposure (Figure 1). Compared to the respective control (C; optimal B supply), a high B supply (B) caused a more prominent reduction in shoot biomass accumulation (Figure 1a) in wheat (3-fold decrease) than in sunflower (2-fold decrease). The reduction in root growth was similar in both species (2.5 times in wheat and about 2.2 times in sunflower, Figure 2b). The application of Si under excessive B supply (Si + B) recuperated the growth of the wheat shoot and root to the level achieved with optimal B supply. The shoot growth of sunflower under high B supply was enhanced by about 40% by the addition of Si, but this was still about 1.5 times lower than with optimal B supply; root growth was not significantly ameliorated by Si addition in sunflower.

Under optimal B supply, sunflower accumulated about 5-fold and 2-fold more B in the shoot and root, respectively, compared to wheat (Figure 2). Compared to the respective control, the high B supply increased the B concentrations in wheat shoot by about 35 times (Figure 2a), while in sunflower this increase was far less pronounced (14-fold, Figure 2b). Root B concentrations did not differ between the crops at the high level of B supply. The application of Si under high B supply led to an approximately 2-fold decrease in the shoot B concentrations in both species; at the same time, the sunflower shoot concentrations were two times higher than those in wheat. The root B levels under high B supply were not affected by the addition of Si in both species.

### 2.2. Compartmentation of B in Leaves

The *in planta* distribution of B between the apoplastic (cell wall) and the symplastic (cytosol including vacuole) compartment was different in wheat (Table 1) compared to sunflower (Table 2). At the optimal B supply (C), a majority (82%, Table 1) of B in the youngest fully expanded leaf was bound to the cell wall (apoplastic fraction), while in sunflower this share was two times lower (Table 2). Wheat responded to the excessive B supply (B) with an unchanged concentration of apoplastic B and dramatically (about 167 times) increased symplastic B (Table 1). In contrast, B compartmentation in sunflower was more moderately affected by high external B supply; cell-wall-bound B increased about 4-fold, and cell sap B concentrations increased about 9-fold (Table 2). The addition of Si at a high B supply (B + Si) also had a different effect on these two species. In wheat, the Si supply ameliorated the excessive B levels in the symplast (decreased B concentration by about 50%), and increased the concentrations of cell-wall-bound B by about 300%, leading to a 4.5-fold increase in the share of apoplastic B (Table 1). In contrast, the addition of Si had no effect on B compartmentation under high B supply in sunflower. Moreover, there existed intrinsic differences in the relative importance of B and Si in the cell wall structure of these two species (Table 3). An increase in external B supply caused about a 10-fold decrease in the cell wall ratio of Si:B in both crops, but wheat consistently contained about 40 times more Si relative to B than for sunflower. On the other hand, the application of Si under high B supply did not change the ratio of Ca:B in the cell walls of either species, which was consistently 3-fold higher in sunflower compared to wheat (Table 4).

### 2.3. Effect of Pulsed Supply of Si on the B Binding Ability of Leaf Cell Walls

Time course experiments with stable isotopes ^10^B and ^11^B showed that the leaf apoplastic binding of B under excess B supply was differently affected by the addition of Si in wheat compared to sunflower (Table 5). During the preculture period in nutrient solution containing non-enriched boric acid for 14 d, isotope distribution reflected the natural abundance of B isotopes; so, the differences in *δ*^11^B values can be considered to be on the level of background noise for both species. Simplified, the more negative values of *δ*^11^B indicate the increased presence of ^10^B, while the less negative values indicate the increased presence of ^11^B (see Section 4). When the precultured –Si wheat plants were exposed to ^10^B-enriched solution in the presence of Si for 3 d, the pronouncedly more negative value of *δ*^11^B indicated that they incorporated more B in the leaf cell wall compared to +Si precultured wheat individuals transferred to Si-free medium. This pattern in wheat was confirmed during the subsequent 3 d in the same group of plants, this time exposed to ^11^B; again, Si-deprived wheat plants under high B stress reacted to the new supply of Si by a pulsed increase in cell-wall-bound B, which however failed to occur when Si supply to the growing plants was withdrawn (Table 5). In contrast, no effect of Si supply on the cell wall capacity to bind B in sunflower leaves was detected during the course of the experiment (Table 5). As the sunflower was growing, B continued to be incorporated in the leaf apoplast; for the first 3 d, the *δ*^11^B values indicated the increased share of ^10^B (^10^B-enriched solution), and for the subsequent 3 d they indicated the increased share of ^11^B (^11^B-enriched solution), irrespective of the presence of Si (Table 5).

## 3. Discussion

Monocots and dicots differ in their B requirement [27], which is in accordance with our findings that (a) excessive B supply caused stronger shoot growth depression in wheat compared to sunflower (by about 67% and 49%, respectively; see Figure 1), and (b) under optimal B conditions, wheat plants accumulated less B than the sunflower plants (Figure 2). Under high B conditions, the concentration of B in the wheat shoot was about two times lower than in sunflower (Figure 2a), whereas the species did not differ in the root B concentrations (Figure 2b). The retention of B in roots was not substantial in wheat genotypes tolerant to high B [28], nor in sunflower plants subjected to a range of excess B supply (400–1600 µM) [29]. In the present study, the ameliorative effect of Si on plant growth under high B supply (Figure 1) and the concomitant lower accumulation of B in the leaves of Si-fed plants (Figure 2) could be attributed to the proposed interactions between B and Si in a nutrient solution, thereby reducing B availability [3,9]. Additionally, competition between silicic and boric acids for the influx-type multifunctional aquaporin NIP2;1, which is also known as a homolog of Lsi1 [30,31], cannot be excluded. The addition of Si to B-stressed plants recuperated root and shoot growth to the level of adequate B supply in wheat, but not in sunflower (Figure 1). Moreover, Si supply significantly decreased symplastic B in wheat leaves (Table 1), which might be due to the enhanced export of B from the symplast to the apoplast via Si-mediated borate efflux transporters such as BOR2 [21] and BOR4 [26]. However, Si supply did not affect the symplastic concentration of B in sunflower leaves (Table 2). Thus, the effects of Si on the expression of borate efflux transporters for enhancements of high B tolerance in the shoots of monocots and dicots are to be further examined. 

The structure and composition of cell walls vary among plant species and tissues within species. Overall, cell walls are formed by cellulose embedded in a network of hemicelluloses, hydroxycinnamic acids, and lignin, with the contribution of pectins and structural proteins [32]. In addition, both B and Si maintain the physical strength of wall structures by cross-linking cell wall polymers [33]. In dicots, B is mainly responsible for the integrity of cell walls, by covalently cross-linking the pectic polysaccharide rhamonogalacturonan II (RGII), whereas in monocots, Si cross-links different cell wall components (e.g., hemicelluloses and glucans), thereby improving their structural stability [11,34]. Therefore, the cell walls of wheat and sunflower also differ in their proportion of pectic substances that complex B. For instance, the concentration of complexed B in the root cell walls is up to 30 μg g^−1^ dry weight in dicotyledonous species, what is several times higher than in graminaceous species [35]. A similar range of the cell-wall-bound B was also found in the leaves of wheat and sunflower under optimal B supply (Table 1 and Table 2). However, wheat leaves showed a greater relative share of apoplastic B (82%) than the sunflower leaves (44%). In wheat under a high B supply, this proportion of leaf apoplastic B dramatically decreased to only 4% (Table 1). However, sunflower leaves still showed a high retention of B in the cell wall (28%), which is in accordance with the study of Dannel et al. [29]. At the same time, the Si supply enhanced cell wall binding potential for B in wheat (18% of relative apoplastic B; see Table 1), but not in sunflower (Table 2). The cell wall binding capacity for B in sunflower leaves was saturated, and so Si did not further extend the binding sites for B as it did in the wheat leaves. The relative share of Si and B in the cell wall (Si:B ratio) was about 10 times lower under high B supply in both wheat and sunflower leaves, being significantly higher in wheat than in sunflower (Table 3). Monocots and dicots differ in their amounts of cell wall components that bind calcium (Ca), which also play structural roles in the cell wall assembly [36,37]. Maintaining a constant ratio of Ca:B in the cell wall of both cultivars, irrespectively of Si treatments (Table 4), indicates that Si did not affect the binding of Ca in the cell wall.

Our findings that the presence of Si continuously extends the cell wall capacity for binding excess B in wheat leaves were further confirmed in the experiment with stable B isotopes (^10^B- or ^11^B-enriched solutions). Wheat plants precultured in –Si nutrient solution supplied with high B (natural abundance of ^10^B and ^11^B) dramatically decreased the δ^11^B value when they were transferred to the +Si solution enriched with ^10^B (Table 5). This clearly indicates that a 3 d supply of Si led to the extension of the cell wall binding sites for B in the Si-accumulating species such as wheat. On the other hand, the cell wall of sunflower leaves also showed a tendency for the continuous binding of ^10^B (e.g., decreased δ^11^B value), but it was unaffected by the Si supply (Table 5). After the further withdrawal of Si from the nutrient solution and the exposure of wheat plants to ^11^B-enriched solution for another 3 d, the B retention potential of the cell wall in both species was slightly increased (see Table 5). However, the presence of Si in the nutrient solution (a second cycle of pulsed Si supply) increased the cell wall binding of B only in the wheat leaves (Table 5). Obviously in sunflower, a species with constitutively higher cell wall retention capacity for B [35], the pulsed supply of Si did not contribute in the extension of the binding sites for B. A continuous supply of Si is needed for a continuous B retention in the leaf cell walls of wheat with lower binding capacity for B, however, it shows only a minor effect in sunflower which is rich in B binding sites (Table 5). Overall, the time course experiment with stable B isotopes showed that the supply of Si under excessive B levels does increase the capacity of the cell wall to bind B during plant growth in a monocot (constitutively higher Si accumulation and lower B requirements), but not in a dicot (constitutively lower Si accumulation and higher B requirements) species, thus reflecting the intrinsic differences in their cell wall structure and composition. A lack of Si leads to the remodeling of the structure and linkages of cell wall components of Si-accumulating species such as grasses, mostly affecting pectins [38]. Since pectic polysaccharides (e.g., RGIIs) are predominantly cross-linked by B [34], a significant extension for the binding of excess B when –Si wheat plants switch to +Si conditions might be attributed to the involvement of B in the recompositing and restructuring of cell walls. The direct replacement of Si with excess B is less possible, since Si can also be strongly bound to the pectin substances rich in galacturonate residues [11]. However, the formation of borate-silicate complexes prior to association with the cell wall matrix cannot be excluded. 

In conclusion, the present results from hydroponic experiments under controlled conditions confirmed our hypothesis that the application of Si differently influences the binding capacity of leaf cell walls for B in wheat and sunflower, which greatly differ in their ability to accumulate these two mineral elements. In wheat, whose capacity to retain B in the leaf cell walls is lower than in sunflower, a continuous supply of Si is crucial for an enhancement of high B tolerance in the shoot. On the other hand, in sunflower, whose cell walls are constitutively rich in galacturonate and bind B, the supply of Si did not contribute significantly in the extension of the B binding sites in leaves. From one perspective, these findings may practically be relevant for a wider application of cropping practices (e.g., crop reside management, crop rotation, intercropping, etc.) besides Si fertilization, to increase rhizosphere Si availability in the sustainable cultivation of wheat in high-B soils which are, in general, low in available Si. 

## 4. Materials and Methods

### 4.1. Plant Material, Growth Conditions and Experimental Design

The seeds of sunflower (*Helianthus annuus* L. cv. Duško) and wheat (*Triticum vulgare* L. cv. Pobeda) were germinated in quartz sand moistened with saturated CaSO_4_ at 25 °C. The 4-day-old seedlings were then transferred to a complete nutrient solution (4 and 20 sunflower and wheat plants, respectively, per 3 L plastic pot) containing the following: (in mM) 0.7 K_2_SO_4_, 0.1 KCl, 2.0 Ca(NO_3_)_2_, 0.5 MgSO_4_, and 0.1 KH_2_PO_4_, and (in µM) 0.5 MnSO_4_, 0.5 ZnSO_4_, 0.2 CuSO_4_, 0.01 (NH_4_)_6_Mo_7_O_24_, and 20 Fe(III)-EDTA. Boron was supplied as H_3_BO_3_ either in optimal (1 µM for wheat and 10 µM for sunflower) or excessive (500 µM for both crops) concentrations. If applied, Si was in the form of monosilicic acid (H_4_SiO_4_) at 1.5 mM; it was freshly prepared by passing Na_2_SiO_3_ through a plastic column filled with cation-exchange resin (Amberlite IR-120, H^+^ form; Fluka, Buchs SG, Switzerland) [39]. The initial pH of Na_2_SiO_3_ stock solution was 11–12, and after passing it through a cation-exchange resin, the pH dropped to about 3. The final concentration of H_4_SiO_4_ in the nutrient solution, determined using the molybdenum blue method [40], was in the range of 1.4–1.5 mM. The plants were grown for 14 d in the following treatments: control (C; optimal B), excess B (B), and excess B with Si supply (B + Si). 

In the additional experiment, the 4-day old plants (wheat and sunflower) were grown with 500 µM B (natural abundance of B) in ether −Si or +Si (1.5 mM) solution for 14 d. Plants were then transferred to ^10^B-enriched solution (500 µM H_3_^10^BO_3_, 99% atom ^10^B; Sigma-Aldrich Inc., St. Louis, MO, USA) following the exclusion of Si from the +Si solution and the addition of 1.5 mM H_4_SiO_4_ to the −Si solution. After 3 d of exposure to high ^10^B-enriched (+/− Si) solutions, the +Si plants were transferred to –Si solution and the –Si plants were transferred to +Si solution and exposed to ^11^B-enriched solution (500 µM H_3_^11^BO_3_, 99% atom ^11^B; Sigma-Aldrich) for another 3 d. 

The pH of the nutrient solutions was adjusted to 6.0 and checked daily. The nutrient solutions were renewed completely every 4 d, unless mentioned separately in the text (additional experiment with B isotopes), and continuously aerated. The plants were grown under controlled environmental conditions with a light/dark regime of 16/8 h, air temperature of 25 °C, photon flux density of 250 µmol m^−2^ s^−1^ at plant height (provided by multispectral led panels; Apollo 8, Cidly Co., Ltd., Shenzhen, China), and a relative humidity of about 70%. Nutrient solutions were renewed every 4 days. 

After the experiments, the plants were harvested and divided in two parts, root and shoot, oven dried at 70 °C for 48 h, and used for total B analyses. In parallel, the youngest fully expanded leaves were used for fractionated B analyses.

### 4.2. Fractionation of B

The major midribs of the youngest fully expanded leaves were removed, and the leaf segments were frozen using liquid nitrogen, then thawed and pressed, and centrifugated at 4000× *g* using Amicon^®^ Ultra-15 Centrifugal Filter Unit (Merck Millipore, Bedford, MA, USA). The supernatant (cell sap) represents the symplastic B fraction. The solid residue was homogenized with 40 mL of deionized H_2_O and centrifuged at 3500× *g*. The pellet was resuspended in 30 mL deionized H_2_O and successively pelleted at 1000× *g* two times. The final pellet (cell wall material) was dried at 65 °C, representing the apoplastic B fraction.

### 4.3. Determination of B and Ca

Dry root, leaf, and cell wall materials (0.2 g) were microwave digested with 3 mL of HNO_3_ and 2 mL of H_2_O_2_ (ETHOS EASY, Milestone Srl, Sorisole, Italy) for 1 h; the cell sap material was evaporated for dryness and dissolved in 5 M HNO_3_. The total concentrations of B (root, leaf, and cell wall materials) and Ca (cell wall material) were determined via inductively coupled plasma optical emission spectroscopy (ICP-OES; Spectro-Genesis EOP II, SPECTRO Analytical Instruments GmbH, Kleve, Germany). 

The concentrations of ^10^B and ^11^B in the cell wall material were determined via inductively coupled plasma mass spectrometry (ICP-MS; Agilent 8900 ICP-QQQ, Agilent Technologies, Inc., Santa Clara, CA, USA) after a final dilution of the samples at 1:20 (*v*/*v*) with deionized H_2_O [41]. The H_3_BO_3_ isotopic standard NIST SRM 951 was used for δ^11^B determination following the below equation:

δ^11^B_sample_ (‰) = [(^11^B/^10^B)_sample_/(^11^B/^10^B)_standard_ − 1] × 1000

### 4.4. Determination of Si

Dry cell wall material (0.2 g) was microwave digested as described above. Samples were diluted with 15 mL deionized H_2_O, transferred into 25 mL plastic flasks, and after the addition of 1 mL concentrated HF were left overnight [40]. The flask volume was adjusted to 25 mL and the concentration of Si was determined via ICP-OES, supplied with an HF-resistant sample introduction system (SPECTRO Analytical Instruments), after a final dilution of the samples at 1:100 (*v*/*v*) with deionized H_2_O.

### 4.5. Statistical Analysis

Data were subjected to analysis of variance using the statistical software Statistica 6 (StatSoft Inc., Tulsa, OK, USA) and means were post hoc compared using Tukey’s test at the 5% significance level (α = 0.05).

## Figures and Tables

**Table 1:** Apoplastic and symplastic concentrations and relative apoplastic share of B in the youngest fully expanded wheat leaves.

Treatments ^1^	B Concentration (µg g^−1^ DW)		Relative Share of Apoplastic B (%)
	Apoplastic	Symplastic	
C	8.2 ± 1.5 b	1.8 ± 0.2 c	82
B	10 ± 1 b	264 ± 22 a	4
B + Si	32 ± 4 a	144 ± 31 b	18

^1^ C (control), 1 µM B; B, 500 µM B; B + Si, 500 µM B + 1.5 mM Si. Data are means of 4 replicates ± SD. Statistically significant differences between treatments for each compartment (*p* < 0.05, Tukey’s test) are indicated by different letters.

**Table 2:** Apoplastic and symplastic concentrations and relative apoplastic share of B in the youngest fully expanded sunflower leaves.

Treatments ^1^	B Concentration (µg g^−1^ DW)		Relative Share of Apoplastic B (%)
	Apoplastic	Symplastic	
C	33.2 ± 4.1 b	41.6 ± 5.0 b	44
B	129 ± 13 a	361 ± 7 a	28
B + Si	131 ± 12 a	367 ± 14 a	28

^1^ C (control), 10 µM B; B, 500 µM B; B + Si, 500 µM B + 1.5 mM Si. Data are means of 4 replicates ± SD. Statistically significant differences between treatments for each compartment (*p* < 0.05, Tukey’s test) are indicated by different letters.

**Table 3:** Relative proportion of Si and B in the cell wall material from the youngest fully expanded leaves of wheat and sunflower grown at 1.5 mM Si.

B Supply	Si:B Ratio	
	Wheat	Sunflower
Optimal	12.38 a	0.29 a
High	1.27 b	0.03 b

Optimal, 1 µM B for wheat and 10 µM B for sunflower; high, 500 µM B. Data are means of 4 replicates. Statistically significant differences between treatments for each crop (*p* < 0.05, Tukey’s test) are indicated by different letters.

**Table 4:** Effect of Si supply (1.5 mM) on the relative proportion of Ca and B in the cell wall material from the youngest fully expanded leaves of wheat and sunflower.

Si Supply	Ca:B Ratio			
	Wheat		Sunflower	
	Optimal B	High B	Optimal B	High B
−	10.0 a	1.3 a	17.5 a	0.5 a
+	10.2 a	1.5 a	17.4 a	0.5 a

Optimal B, 1 µM B for wheat and 10 µM B for sunflower; high B, 500 µM B. Data are means of 4 replicates. Statistically significant differences between Si and B treatments for each crop (*p* < 0.05, Tukey’s test) are indicated by different letters.

**Table 5:** Effect of pulsed Si supply on δ^11^B changes in the cell wall material isolated from the youngest fully expanded leaves of wheat and sunflower at excess B.

Exposure Time (d)	B Isotope	Treatments	δ^11^B (‰)	
			Wheat	Sunflower
14	^10^B/^11^B (natural)	−Si	5	9
		+Si	17	11
3	^10^B (enriched)	+Si ← −Si	−705	−646
		−Si ← +Si	−310	−625
3	^11^B (enriched)	−Si ← +Si	−711	−573
		+Si ← −Si	−322	−591

Plants were precultured in either –Si or +Si (1.5 mM) nutrient solution with 500 µM non-enriched B for 14 d. The −Si plants were then transferred to +Si solution and the +Si plants were transferred to −Si solution. After 3 d of exposure to 500 µM enriched ^10^B, the −Si solution was replaced with +Si solution and *vice versa*, and the plants were further exposed to 500 µM enriched ^11^B for another 3 d. Data are means of 4 replicates.

## Data Availability

All of the relevant data are presented in the tables and figures of this paper.
